# Deletion of MicroRNA-144/451 Cluster Aggravated Brain Injury in Intracerebral Hemorrhage Mice by Targeting 14-3-3ζ

**DOI:** 10.3389/fneur.2020.551411

**Published:** 2021-01-12

**Authors:** Xiaohong Wang, Yin Hong, Lei Wu, Xiaochun Duan, Yue Hu, Yongan Sun, Yanqiu Wei, Zhen Dong, Chenghao Wu, Duonan Yu, Jun Xu

**Affiliations:** ^1^School of Medicine, Yangzhou University, Yangzhou, China; ^2^Jiangsu Key Laboratory of Experimental & Translational Non-coding RNA ResearchNoncoding RNA Center, Yangzhou University, Yangzhou, China; ^3^Jiangsu Co-innovation Center for Prevention and Control of Important Animal Infectious Diseases and Zoonoses, Yangzhou University, Yangzhou, China; ^4^National Center for Clinical Research of Nervous System Diseases, Beijing Tiantan Hospital, Capital Medical University, Beijing, China; ^5^Department of Neurosurgery, Affiliated Hospital of Yangzhou University, Yangzhou, China; ^6^Department of Neurology, Zhangjiagang City First People's Hospital, Zhangjiagang, China; ^7^Department of Neurology, Peking University First Hospital, Beijing, China; ^8^Department of Neurology, Beijing Tiantan Hospital, Capital Medical University, Beijing, China

**Keywords:** microRNA-451, intracerebral hemorrhage (ICH), inflammation, 14-3-3ζ, mice

## Abstract

This study aims at evaluating the importance and its underlying mechanism of the cluster of microRNA-144/451 (miR-144/451) in the models with intracerebral hemorrhage (ICH). A model of collagenase-induced mice with ICH and a model of mice with simple miR-144/451 gene knockout (KO) were used in this study. Neurodeficits and the water content of the brain of the mice in each group were detected 3 days after collagenase injection. The secretion of proinflammatory cytokines, such as tumor necrosis factor α (TNF-α) and interleukin 1β (IL-1β), as well as certain biomarkers of oxidative stress, was determined in this study. The results revealed that the expression of miR-451 significantly decreased in the mice with ICH, whereas miR-144 showed no significant changes. KO of the cluster of miR-144/451 exacerbated the neurological deficits and brain edema in the mice with ICH. Further analyses demonstrated that the KO of the cluster of miR-144/451 significantly promoted the secretion of TNF-α and IL-1β and the oxidative stress in the perihematomal region of the mice with ICH. In addition, the miR-144/451's depletion inhibited the regulatory axis' activities of miR-451-14-3-3ζ-FoxO3 in the mice with ICH. In conclusion, these data demonstrated that miR-144/451 might protect the mice with ICH against neuroinflammation and oxidative stress by targeting the pathway of miR-451-14-3-3ζ-FoxO3.

## Introduction

As a devastating stroke subtype, intracerebral hemorrhage (ICH) is related to poor prognosis and high disability (1). Serious sequela of survivors and the lack of effective clinical treatments bring health burdens to the patients with ICH and their families (2). Currently, many treatments need to be explored in preclinical and clinical researches of ICH, but there is no effective treatment (3). Thus, it has been suggested that the development of novel and effective therapeutic treatments for ICH remains an important area of preclinical researches.

The pathogenic processes of ICH include the primary and secondary brain injuries. The primary brain injuries stem from rapid hematoma and then develop various biological effects including oxidative stress and neuroinflammation, leading to secondary brain injuries. Neuroinflammation cascade, involving microglia activation, secretion of proinflammatory cytokines, and oxidative stress, accelerates neuronal death and edema exacerbation after ICH (4, 5). Therefore, the identification of mechanisms of inflammation following ICH may provide promising strategies for brain injuries and poor prognosis.

As non-coding RNAs, microRNAs (miRNAs) inhibit the posttranscription of the target genes through binding to their 3′ untranslated region (6–8). miRNAs have been proposed to be novel biomarkers and regulatory molecules in the diagnosis and treatment of ICH (9). Increasing studies show that miRNAs are involved in the pathogenesis and development of ICH in preclinical researches (10, 11). For example, it is found that miR-126-3p mimic administration significantly alleviated the neurological deficits of the mice with ICH and inhibited BBB disruption and cerebral edema by targeting the pathway of PIK3R2-mediated PI3K/Akt in the perihematomal area (10). Qu Xin et al. reported that miR-146a mimic injection obviously improved the motor function and alleviated the brain edema, suppressed the secretion of proinflammatory cytokines tumor necrosis factor α (TNF-α) and interleukin 1β (IL-1β), and suppressed the oxidative stress around hematomas of the mice with ICH (11).

miR-144 and miR-451 (miR-144/451) with high conversion in different species were processed from a single RNA precursor transcript and abundantly expressed in erythrocyte precursors (12). miR-144/451 facilitated terminal maturation of erythrocyte precursors and protected from oxidant stress through down-regulating 14-3-3ζ in erythroid cells (13). Knockdown of miR-144/451 triggers the risk of lung, gastric carcinoma, and bladder cancer, suggesting the cluster as a potential tumor suppressor (14–16). It has been found that miR-451 could suppress the microglia-mediated inflammation in chronic inflammatory pain by targeting TLR4 (17). Liu et al. showed that miR451 could protect neurons against oxidative stress in an oxygen and glucose deprivation/reoxygenation (OGD/R) cell model through inhibiting its target protein-CUGBP Elav-like family member 2(CELF2) (18). Fu et al. reported a negative association between miR-451 levels in blood of ischemic stroke patients and National Institutes of Health Stroke Scale scores and infarct volume (19). Little is known on the behavior of miR-451 in the development of post-ICH complications, namely, edema and neuroinflammation.

Thus, the work reported herein aims to address the effects of the cluster of miR-144/451 on behavioral deficits, neuroinflammation, and oxidative stress around hematomas in mice model with ICH. This work first evaluated the importance of the cluster of miR-144/451 during the occurrence of ICH using the mice with miR-144/451 gene knockout (KO).

## Materials/Methods

### Animals/the Establishment of Model With ICH

#### Animals

Gene KO mice lacking miR-144/451 cluster (referred to as KO) were defective in miR-144/451 expression in all kinds of tissue as previously described (13). The KO mice were obtained from University of Pennsylvania and kept in a standard pathogen-free and quiet room. Wild-type mice with same background (C57BL/6J) were used for the control. The animals were divided into four groups (*n* = 15) randomly: a sham, an ICH, an ICH + miR-144/451 KO (ICH + KO), and a sham + miR-144/451 KO group. The protocols used in this study were with the approval of the Institutional Animal Care and Use Committee and the Animal Ethics Committee of Yangzhou University [SYXK (Su) IACUC 2017-0045]. These animals were all kept in a room under a 12-h light/dark cycle at 23°C ± 1°C with free access to water/foods.

#### The Establishment of the Model of ICH

These animals were anesthetized by intraperitoneal administration of 4% chloral hydrate and then fixed onto a stereotaxic frame. We then located the right caudate putamen using the stereotactic coordinates (coordinates related to bregma, anteroposterior (AP), mediolateral (ML), and dorsoventral (DV), are 0.2, 3.7, and 3.8 mm, respectively) according to the previous description (20). Type IV collagenase (0.05 U) (Sigma–Aldrich Co., St. Louis, MO, USA) was injected using a syringe. The needle had been kept in the brain for 5 min additionally to prevent back-leakage. The mice in the sham group and KO group were injected with the same volume of normal saline.

### Measurements of Neurological Outcome

During the neurological function tests, all of the animals were kept from any stress, and their well-being was monitored. All of the behavioral training and tests were carried out in a quiet room at a fixed time point everyday by at least two experimenters blinded to the behavioral test.

#### Cylinder Test

The cylinder test was performed by an examiner blinded to the experiment for the measurement of spontaneous forelimb use. The mice (*n* = 9 per group) had been put in a transparent cylinder and videotaped for 5 min. A forelimb was placed on the wall for the first time, and the later movements along the wall were counted. The percentage of the affected limb use was calculated.

#### Rotarod Test

Motor coordination of the mice was investigated using the rotarod test. In brief, all animals (*n* = 9 per group) had been trained by an examiner blinded to the experiment, at the speed of 4–30 revolutions/min (rpm) for 5 min, three times per day, for 3 days before the induction of ICH. The mice that fell off the rod were put back with minimal disturbance. The mice that had not achieved stable performances after training were kicked from the test. After the operation of ICH, the mice were subjected to three trials at the speed of 40 rpm, with a 5-min rest between each trial. The latency to fall of each trial was recorded. The mice staying on the rod for more than 300 s were removed, and the latency to fall was recorded as 300 s.

#### Corner Turn Test

The mice of all groups (*n* = 9 per group) were put in a 30° corner, and they will quit through turning to the left side or to the right side. Only those turnings with full rearing along either wall were recorded. After the operation of ICH, the mice tend to turn to the ipsilateral to the damages (21). The test had been performed for 10 times, at an interval of 1 min, and the right turns' percentage was recorded.

#### Water Content of the Brain

It was determined 3 days after the surgery. Brains (*n* = 5 per group) were collected and dissected into the ipsilateral and contralateral cerebral hemispheres on ice. The wet weight of each cerebral hemisphere was measured using an electric analytic balance. The brain tissues had been dried at 120°C for 24 h until there was no decrease of the weight, and the dry weight was measured. The water content of the brain = (wet weight – dry weight)/wet weight × 100%.

### Nissl Staining and Immunohistochemical Staining

The mice were transcardially perfused with normal saline and paraformaldehyde (4%) 3 days after the operation, and their brains were isolated and dehydrated in 15, 20, and 30% sucrose. Then, the brains were cut with a freezing microtome to collect coronal sections with the thickness of 25 μm. Nissl staining was conducted using a cresyl violet (C9140-1; Solarbio) staining kit following the manufacturer's instructions (Solarbio, China). Immunohistochemical staining for glia fibrillary acidic protein (GFAP) (*n* = 5 per group) was conducted for detecting neuroinflammation. Brain sections were soaked in 0.25% Triton X-100 and 3% H_2_O_2_, blocked in normal goat serum (5%), and then, they had been incubated at 4°C overnight with the primary antibodies anti-GFAP (1:800; Abcam), anti–IL-1β (1:200; Abcam) and FOXO3a (1:200; Abcam), followed by anti–rabbit immunoglobulin G (H + L) (1:200, AS003; Abcam) of horseradish peroxidase goat. Sections were developed with a DAB kit (Zymed Laboratories Inc., San Francisco, CA, USA). The digitized images were obtained with a microscope (Axioplan 2, Zeiss, Oberkochen). The GFAP and IL-1β-positive neurons were counted by means of the optical fractionator and a computer-assisted stereological Olympus Toolbox system. The brain section was first delineated using a 4× objective. A square grid of 150 × 150 μm was randomly superimposed with and a 100 × 100-μm square dissector counting chamber placed on the counting area of the image and moved through all of the counting areas until the whole section was finished.

For the double-immunofluorescence staining, sections were rinsing with phosphate-buffered saline (PBS) for three times and were blocked with normal goat serum for 1 h at room temperature and then incubated with rabbit anti-NeuN (1:400, Abcam) and mouse anti-Iba1 (1:200, Abcam) overnight at 4°C. Then slices were incubated with Alexa Fluor 488–conjugated secondary antibodies (goat anti–mouse, 1:400, GB25301; goat anti–rabbit, 1:400, GB25303; Servicebio) for 1 h at room temperature. The brain slices were stained with DAPI for 5 min. The expression of NeuN and Iba1 in neuron was captured under a fluorescence microscope using Image-Pro Plus 6.0 (Media Cybernetics, Silver Spring, MD, USA). The Iba1-positive cells were counted and presented as cells/mm^2^.

### TUNEL Assay

TUNEL staining was performed to detect neuronal apoptosis in mice of different groups on day 3 after ICH (*n* = 5). Brain sections were washed with PBS and incubated with 20 μg/mL protease K solution. Then brain sections were washed and incubated with the TUNEL reaction mix. TUNEL-positive cells were imaged and calculated under a fluorescence microscope. Data are presented as TUNEL-positive cells/mm^2^.

### Extraction of RNA and Reverse Transcription–Quantitative Polymerase Chain Reaction

The brain tissue and blood were lysed, and RNA was extracted with Trizol reagent (Invitrogen, USA) and was purified with a purification kit (Thermo Fisher scientific). For detection of the levels of mRNA, 1 μg RNA was reverse transcribed into cDNA using the PrimeScript™ II First-Strand cDNA synthesis kit (Takara Bio, Inc., Dalian, Japan) while using the Mir-XTM miRNA First-Strand Synthesis Kit (Takara Bio, Inc., Dalian, Japan) for miR-144 and miR-451, according to the manufacturer's instructions. The cDNA was amplified with SuperScript III Reverse Transcriptase (Invitrogen, USA). Transcripts were detected with PrimeScript™ RT Master Mix (Takara Bio, Inc., Dalian, Japan) in accordance with the instructions of the manufacturer. The genes' mRNA levels were standardized to the housekeeping gene GAPDH; U6 was used as internal control of miR-144 and miR-451. All genes' primer sequences are presented in Table 1. Relative levels of miRNA and mRNA were calculated using 2^−ΔΔCT^ method. All the reactions were run in triplicate.

**Table 1:** The sequences of the primers.

**Name of primers**	**Sequence of primers**
Forward-Sod1	5′−TGAAGAGAGGCATGTTGGAG−3′
Reverse-Sod1	5′−CCACCTTTGCCCAAGTCATC−3′
Forward-Sod2	5′−TCATGCAGCTGCACCACAGC−3′
Reverse-Sod2	5′−CCATTGAACTTCAGTGCAGG−3′
Forward-Cat	5′−TCACTGACGAGATGGCACAC−3′
Reverse-Cat	5′−CTGACTCTCCAGCGACTGTG−3′
Forward-Gpx1	5′−CTCAAGTACGTCCGACCTGG−3′
Reverce-Gpx1	5′−TGTCGATGGTACGAAAGCGG−3′
Forward-GAPDH	5′−AAGGTGAAGGTC GGAGTCAAC−3′
Reverse-GAPDH	5′−GGGGTCATTGATGGCAACAATA−3′
MiR-144	5′−UACAGUAUAGAUGAUGUACU−3′
MiR-451	5′−AAACCGTTACCATTACTGAGTT−3′
U6	5′−CGCTTCGGCAGCACATATAC−3′

### Western Blot

Perihematomal striatum was rapidly dissected and homogenized in ice-cold lysis buffer 3 days after the operation of ICH. The brain extracts were loaded on 10 or 12% sodium dodecyl sulfate–polyacrylamide gel electrophoresis and transferred onto nitrocellulose membranes (Millipore, Massachusetts, USA), which had been blocked with non-fat milk (5%) for 1 h and then had been incubated with primary antibodies against 14-3-3ζ, 14-3-3-β, 14-3-3-θ, 14-3-3-pan (Chemicon International), FoxO3 (Millipore), and β-actin (Abcam Plc, Cambridge, UK) at 4°C overnight. Then, the samples had been incubated with the secondary antibody for 2 h at room temperature, and then the protein bands were visualized with super-enhanced chemiluminescence, later the analysis of the bands' density was performed using ImageJ (National Institutes of Health, Bethesda, MD, USA).

### Enzyme-Linked Immunosorbent Assay

Perihematomal striatum had been put in the lysis buffer and centrifuged at 4°C for 30 min to collect the supernatant 3 days after the operation of ICH operation. The levels of TNF-α and IL-1β were determined using the commercial enzyme-linked immunosorbent assay kits (PEPROTECH, USA). According to the manufacturer's instruction, homogenates of individual mice were placed into the 96-well plates, and then, the corresponding primary antibodies were put and then had been incubated overnight at 4°C. The primary antibody was removed, and the sample was blocked with 1% bovine serum albumin. The secondary antibody was put and had been incubated for 1 h. The measurement of the optical density was performed with a microplate reader at 450 nm.

### SOD and GSH-Px Activity and MDA Content Estimation

The perihematomal tissues were dissected quickly, homogenized, and diluted using precooled 0.01 M PBS (*n* = 4 per group). MDA concentrations were spectrophotometrically detected at 532 nm according to the instructions of the Nanjing Jiancheng Kit. Brain tissue homogenate was centrifuged at 1,500*g*, 10 min, at 4°C, to collect the supernatant for analyzing the activities of superoxide dismutase (SOD) and glutathione peroxidase (GSH-Px). GSH-Px activity was determined by absorbance at 412 nm, while SOD activity at 560 nm according the protocols of the commercial test kits (Jiancheng, Nanjing).

### Collection of Blood Samples

The peripheral blood samples were obtained from the patients with acute cerebral hemorrhage and the healthy controls who were registered in Northern Jiangsu People's Hospital, which Medical Ethics Committee approved the collection of all blood samples. The serum samples of the patients (*n* = 20) were collected 12 h after admission, and the serum samples of the healthy controls (*n* = 18) were collected 1 day after admission. The serum was collected through centrifuging at 1,500 g/min for 10 min.

### Statistical Analysis

All data were analyzed using version 19.0 of SPSS Software (SPSS Inc., Chicago, IL, USA). The data are shown as the mean ± standard error of the mean. The comparisons between each group were analyzed by two-way analysis of variance through *post hoc* Newman–Keuls test. *P* < 0.05 was considered as statistically significant.

## Results

### Time Course of the Expression of miR-144/451 in Perihematomal Area Following ICH

**Figure 1:**
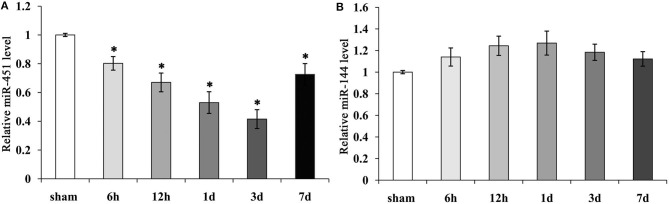
Expression of miR-144 and miR-451 in the peri-hematomal area following ICH. **(A)** miR-451 gradually declined significantly from 6 h to 3 days, then recover at 7 days following ICH, Sham group was used as a control. **(B)** miR-144 increased slightly from 6 h to 1 day, then decreased to that of sham-operation group. Five mice were included in each group. Data were presented as the mean ± standard deviation (*n* = 9). *p < 0.05 vs. the sham group. miR, microRNA; ICH, intracerebral hemorrhage.

To detect the levels of miR-144/451 after ICH, we checked their expressions in the perihematomal area obtained from the mice with ICH. The mRNA levels of miR-144/ 451 were analyzed through reverse transcription–quantitative polymerase chain reaction (qRT-PCR) analysis, and it was found that compared with the sham group, miR-451 gradually declined from 6 h to 3 days and then recovered at 7 days following ICH (*P* < 0.05, Figure 1A). The level of miR-144 increased slightly from 6 h to 1 day, followed by a decrease to that of the sham-operation group with *P* > 0.05, shown in Figure 1B.

### KO of miR-144/451 Deteriorated the Neurological Deficits and Brain Edema of ICH Mice

**Figure 2:**
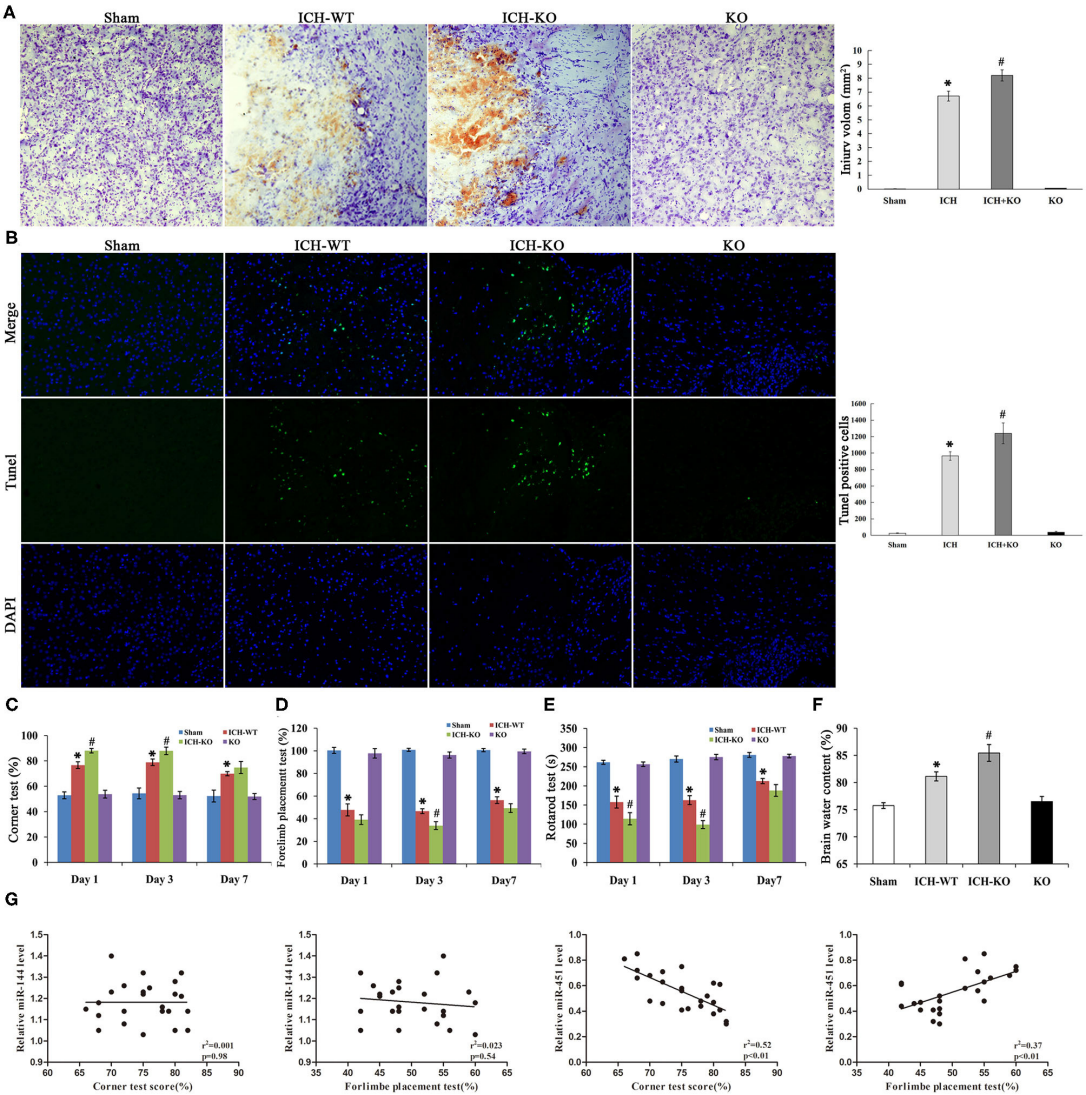
Knockout of miR-144/451 cluster aggravated neurological function and brain injury of ICH mice. **(A)** Representative Nissl stained images of hematoma area on day 3 after ICH (*n* = 5). **(B)** Representative images of TUNEL (green) positive neurons in peri-hematomal area of mice in different groups (*n* = 5). **(C)** Corner tests were performed on days 1, 3 and 7 (*n* = 9). **(D)** Forelimb placement tests were performed on days 1, 3 and 7 (*n* = 9). **(E)** The time on the rod in the rotarod test on days 1, 3 and 7 (*n* = 9). **(F)** Brain water content was measured on day 3 after ICH (*n* = 5). **(G)** Correlations between miR-144, miR-451 levels and behavior tests using the Pearson correlation test. Data were presented as the mean ± standard deviation. *p < 0.05 vs. the sham group; #*p* < 0.05 vs. the ICH group. miR, microRNA; ICH, intracerebral hemorrhage; TUNEL, terminal deoxynucleotidyl transferase dUTP nick end labeling.

**Figure 3:**
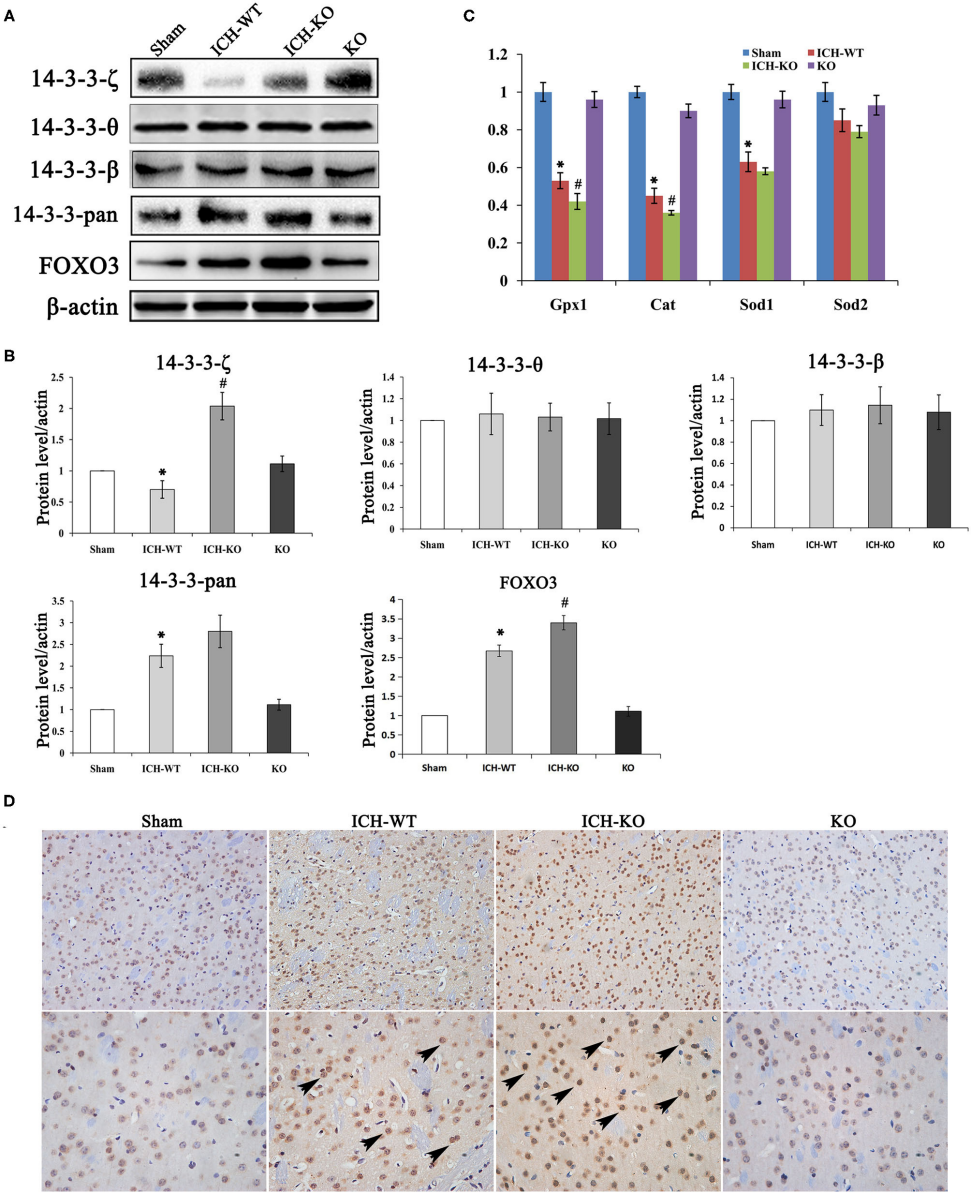
miR-144/451 cluster regulated the expression of 14-3-3ζ and FoxO3 in ICH mice. **(A,B)** The protein expression of 14-3-3ζ, 14-3-3-β, 14-3-3-θ, 14-3-3-pan, Fox3 in the peri-hematomal area of mice in different groups. **(C)** The mRNA levels of genes in the downstream of 14-3-3ζ, including cat, gpx1, sod1, and sod2 in different groups. **(D)** Representative microphotographs showing FoxO3-positive cells in peri-hematomal area on day 3 after ICH. *p < 0.05 vs. the sham group; #*p* < 0.05 vs. the ICH group. miR, microRNA; ICH, intracerebral hemorrhage; FoxO3, forkhead box.

Next, we applied the model of miR-144/451 KO to establish the mice model with ICH to learn more about the importance of the cluster of miR-144/451 after ICH. The corner test, rotarod test, and cylinder test were used for testing neurological deficits in this study. The mice with ICH exhibited significant neurological damages 24 h after ICH, indicating that the model with ICH was established successfully. According to Figure 2A, Nissl staining showed a hematoma area at 3 days after the operation of ICH, and the hematoma area expanded in the ICH-KO group through comparing with the mice with ICH (*P* < 0.05, Figure 2A). The TUNEL assay was carried out to determine the neuronal apoptosis in the perihematomal area of different groups (Figure 2B). The number of TUNEL-positive cells increased significantly in the perihematomal area of the ICH mice (Figure 2B). KO of miR-144/451 cluster significantly aggravated the neuronal apoptosis in ICH mice (*P* < 0.01, Figure 3B).

Compared with the ICH group, the time spent on the rod and frequency of the placements of the left paw were significantly reduced while the right turns of the mice with ICH increased through comparing with the sham group from day 1 after ICH, with *P* < 0.05 shown in Figures 2C–E, and all the neurological parameters were exacerbated in the KO + ICH group obviously with *P* < 0.05 shown in Figures 2C–E, suggesting that the KO of miR-144/451 worsened neurological damages in ICH.

As brain edema is an important biological event in brain injuries after ICH, we determined the water content of the brain of the mice using the wet/dry method. The water content of the brain of the mice was significantly increased after the operation of ICH through comparing with the sham group, which was exacerbated in the mice with miR-144/451KO ICH (*P* < 0.05, Figure 2F).

The Pearson correlation test was applied to analyze the association of the expression levels of miR-144/451 and the corner test and cylinder test. miR-451 revealed a positive correlation with frequency of the placements of the left paw (Figure 2G, *r*^2^ = 0.37, *P* < 0.01) and a negative correlation with right turns (Figure 2G, *r* = 0.52, *P* < 0.01) without any significant correlation between the level of miR-144 and the behavior test (Figure 2G, *p* > 0.05). This indicated that miR-451, not the miR-144, might play the main regulatory role in the brain injuries after ICH.

### KO of miR-144/451 Exacerbated Hemin-Induced Inflammatory Response

**Figure 4:**
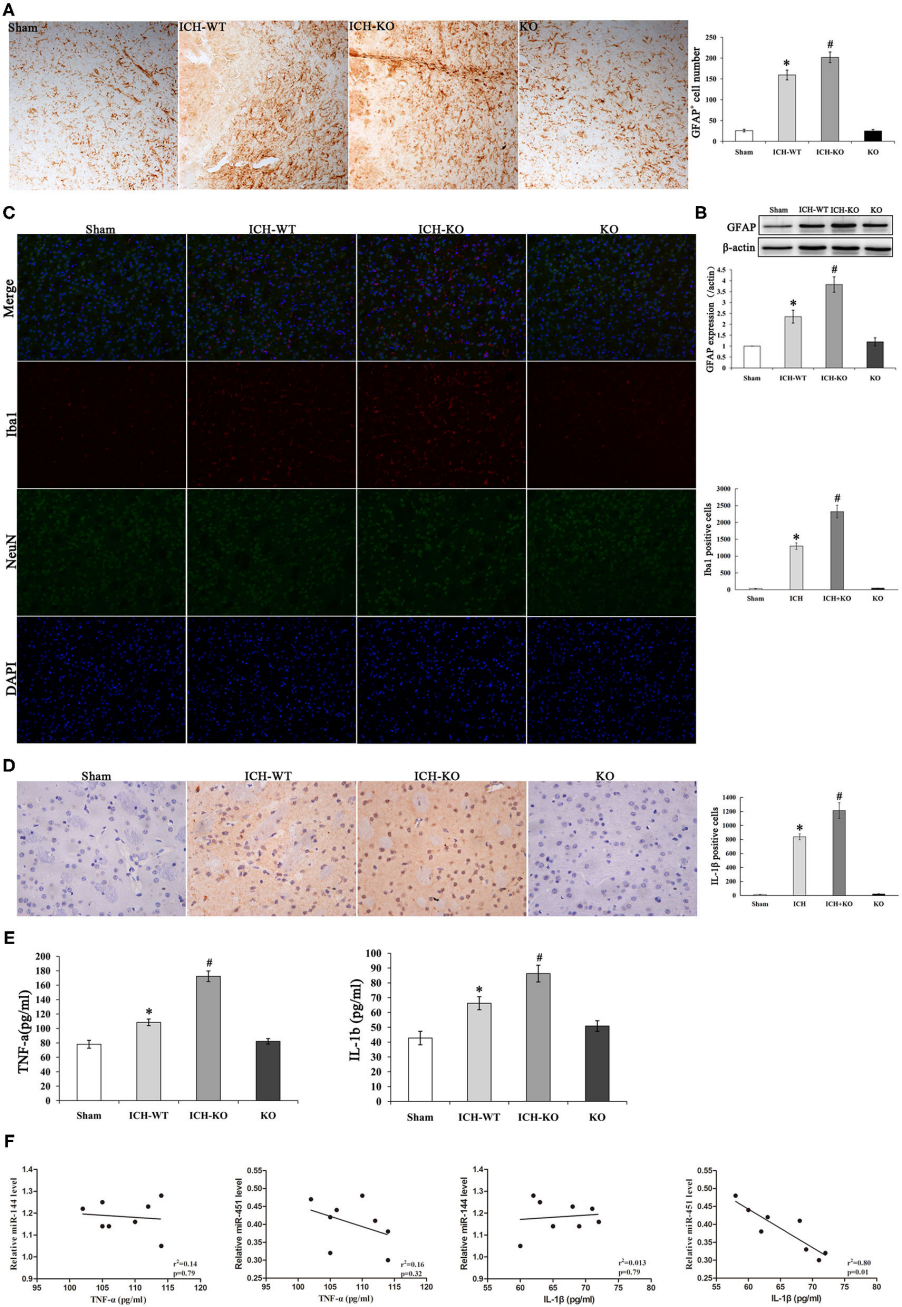
Knockout of miR-144/451 cluster exacerbated neuroinflammation and promoted pro-inflammatory cytokines after ICH. **(A)** Immunohistochemistry for GFAP in peri-hematomal area of mice in different groups (*n* = 5). **(B)** Western Blotting for GFAP in brain of different groups. **(C)** Representative images of double immunofuorescence labeling Iba1 (red) and neuronal nuclei (NeuN)-positive neurons (green) in peri-hematomal area of mice in different groups. **(D)** Representative microphotographs showing IL-1β-positive cells in peri-hematomal area on day 3 after ICH. **(E)** Elisa assays for TNF-α and IL-1β in perihematomal area of different groups (*n* = 5). **(F)** Correlations between miR-144, miR-451 levels and pro-inflammatory cytokines using the Spearman's correlation test. Data were presented as the mean ± standard deviation. *p < 0.05 vs. the sham group; #*p* < 0.05 vs. the ICH group. miR, microRNA; ICH, intracerebral hemorrhage; GFAP, glial fibrillary acidic protein; TNF-α, tumor necrosis factor-α; IL-1β, interleukin-1β; Iba1, Ionized calcium binding adaptor molecule 1; NeuN, neuronal nuclei.

An increasing evidence indicates that inflammatory response is crucial during the development of ICH. ICH activated the GFAP-positive astrocytes and protein level around the hematoma, and miR-144/451 KO markedly exacerbated the number of GFAP^+^ neurons (*P* < 0.05, Figures 4A,B). Activation of microglia also contributes to neuronal injury after ICH. We performed the double-immunofluorescence labeling Iba1 (red) and NeuN (green) to detect the activation of microglia in the perihematomal area of mice. Results showed that the activated microglia is markedly increased in mice after ICH operation; miR-144/451 KO obviously increased the number of Iba1-positive neurons in ICH mice (*P* < 0.05, Figure 4C). We also investigated the secretion of proinflammatory cytokines and found that the depletion of miR-144/451 markedly upregulated the levels of TNF-α and IL-1β in the brains of the mice with ICH (*p* < 0.05, Figures 4D,E). Spearman correlation analysis was applied to detect the correlation between the expression of miR-144/451 and the levels of TNF-α and IL-1β. It presented a negative correlation between the expression of miR-451 and the levels of TNF-α and IL-1β with *P* < 0.01 shown in Figure 4F without a significant correlation between the expression of miR-144 and levels of the TNF-α and IL-6 (*p* > 0.05, Figure 4F).

### KO of miR-144/451 Promoted Oxidative Stress in ICH Mice Brain

**Figure 5:**
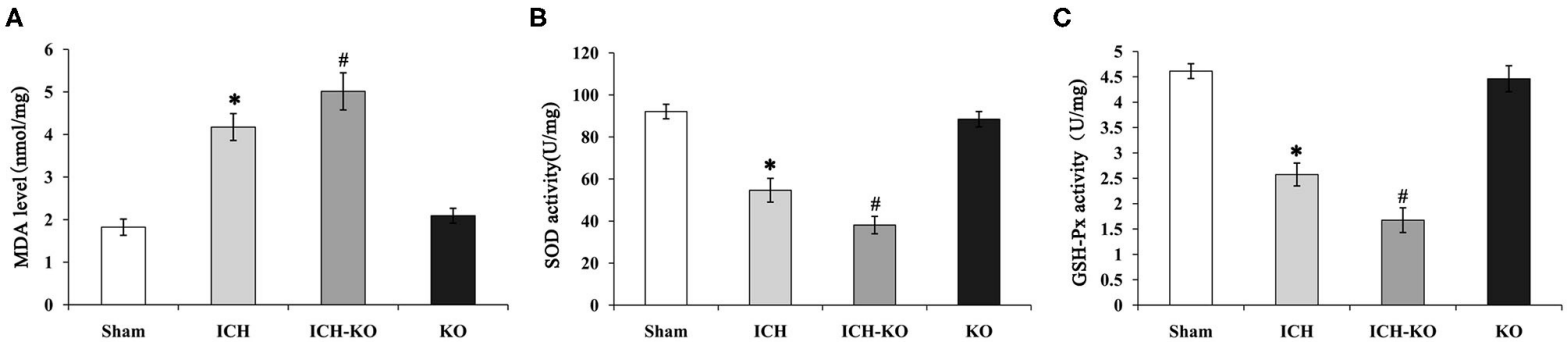
Knockout of miR-144/451 cluster promoted oxidative stress in ICH mice. 3 days after ICH induction, the levels of oxidative stress biomarkers, including **(A)** MDA, **(B)** SOD, and **(C)** GSH-Px were measured in the peri-hematomal area of mice in different groups. Data were presented as the mean ± standard deviation (*n* = 4–5). *P < 0.05 vs. the Sham group; #*P* < 0.05 vs. the ICH group. miR, microRNA; ICH, intracerebral hemorrhage; MDA, malondialdehyde; SOD, superoxide dismutase; GSH-Px, glutathione peroxidase. *p < 0.05 vs. the sham group; #*p* < 0.05 vs. the ICH group.

It is well-known that excessive oxidative stress promotes neuronal injuries in models with ICH (20). This study detected whether the cluster of miR-144/451 could modulate the hemin-induced oxidative stress. We detected the levels of biomarkers of oxidative stress, namely, SOD and MDA, as well as GSH-Px in the perihematomal area. The results showed that the levels of MDA increased significantly, while the activities of SOD and GSH-Px decreased significantly in the mice with ICH through comparing with the sham group, and the depletion of the cluster of miR-144/451 in the mice with ICH promoted these changes significantly (Figures 5A–C, *p* < 0.05). As a previous study reported, miR-451 could suppress oxidative stress by targeting 14-3-3ζ, which promoted the expression of two antioxidant genes-cat and gpx. We also detected some oxidative stress genes, including cat, gpx1, sod1, and sod2. The results revealed that the FoxO3-regulated antioxidant gene cat, gpx1, and sod1 were down-regulated significantly in the mice with ICH through comparing with the sham group, *p* < 0.05, shown in Figure 3C, and the depletion of miR-144/451 markedly worsened the decreasing of Gpx1 and cat, *p* < 0.05, shown in Figure 3C. The levels of SOD2 under the operation of ICH and the depletion of miR-144/451 had no significant change.

### miR-144/451 Repressed the miR-451/14-3-3ζ Axis Pathway in Mice With ICH

Our previous studies have revealed that miR-451 suppresses oxidative stress through targeting the axis of the miR-451 and 14-3-3ζ as well as FoxO3 (13). To determine whether the axis of the miR-451 and 14-3-3ζ as well as Foxo3 is involved in ICH, this study determined the protein levels of 14-3-3ζ and Foxo3. As presented in Figures 3A,B, after the operation of ICH, the protein levels of 14-3-3ζ down-regulated, while Foxo3 up-regulated in the brains of the mice with ICH through comparing with the sham group. The depletion of miR-144/451 significantly up-regulated the expression of 14-3-3ζ and Foxo3 compared with the mice with ICH (Figures 3A,B; *p* < 0.05). KO of the cluster of miR-144/451 did not affect the other subtypes of 14-3-3 protein in mice with ICH. We also detected the expression and location of FoxO3 in the area around the hematoma, as presented in Figure 3D, the FoxO3 transported from cytoplasm to nucleus after ICH operation; KO of miR-144/451 promoted this procession obviously.

### Circulating Levels of miR-144/451 of the Patients With ICH

**Figure 6:**
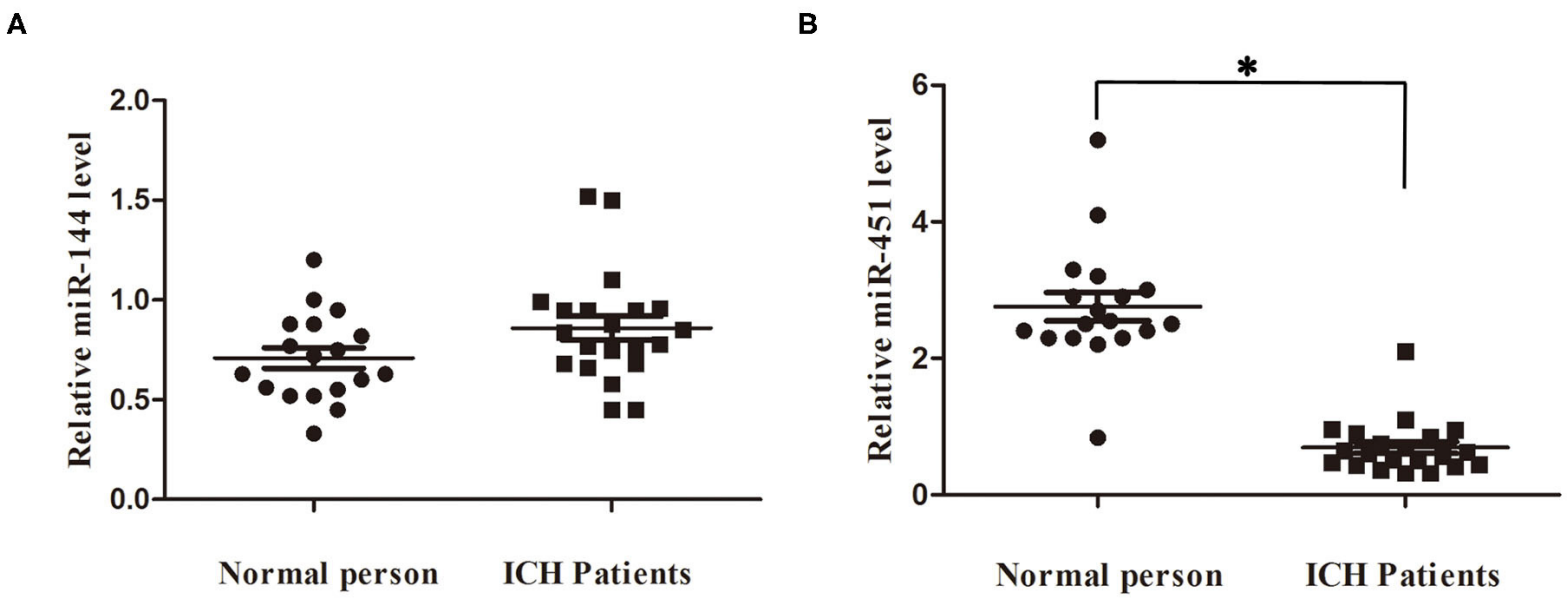
Serum miR-144 and miR-451 was elevated in ICH patients. **(A)** miR-451 declined significantly at 7 days after ICH compared with control people. **(B)** miR-144 showed no significantly change at 7 days after ICH compared with control people. *p < 0.05 vs. the sham group. miR, microRNA; ICH, intracerebral hemorrhage.

We detected the miR-144/451's mRNA levels of the patients with ICH and the healthy controls using the qRT-PCR assay. The clinical characteristics of the ICH patients and control group were described in Table 2 (19). As shown in Figure 6A, circulating miR-451 decreased significantly (*p* < 0.05), while the miR-144 increased to a lesser extent (*p* > 0.05) in the patients with ICH compared with the controlled patients.

**Table 2:** Clinical characteristics of the ICH patients and control group who were used for analyzing the expression of the miR-144 and miR-451 (19).

	**ICH patients**	**Healthy control**	***P*-value**
Ethnicity, (%)	100%	100%	1
Age, (years, mean + SD)	59.8 ± 7.4	62.5 ± 4.2	0.89
Sex, male, (%)	63%	56%	0.53
Hypertension, (%)	56%	40%	0.72
Diabetes, *n*	2	3	0.42
Body mass index, (kg/m^2^, mean + SD)	26.6 ± 8.4	23.5 ± 4.1	0.36
Hours since ICH, (h, mean + SD)	40.6 ± 7.4	N/A	<0.001

## Discussion

ICH, accounting for 10–30% of all stroke types, is one of the major health burdens worldwide (22). ICH survivors frequently suffer from left hemiplegia, speech disorders, and vascular cognitive impairments. To date, there is no effective clinical treatment to alleviate ICH and post-ICH complications (2). Accumulating evidences showed that various miRNAs were identified as biomarkers for diagnosing and the progression of neurological diseases (miR-21-P) (8–11).

miR-144/451 has been reported to be involved in the development of various diseases. Previously, it was found that the miR-451's knockdown in the zebra fish's embryos impaired erythroid maturation significantly (13). Several groups reported that miR-144/451 was critical for suppressing cell survival in several kinds of cancers, including lung cancer, gastric cancer, and so on (14–18). For the neurological diseases, miR-144 elevated in the early stage of AD patients and induced the depletion of ADAM10, which forced metabolism of amyloid β-peptide (Aβ) to protect the brain. Overproduced Aβ recruited the transcription factor AP-1 and GATA and consequently promoted the expression of miR-144, forming the vicious circle (23). Therefore, we hypothesized that miR-144/451 might be involved in the neuronal injuries post the ICH conditions. In the present study, collagenase IV significantly suppressed the expression of miR-451 while just increasing the level of miR-144 slightly in the perihematomal area. Besides, the KO of miR-144/451 aggravated the motor deficits, neuronal apoptosis, and the brain edema significantly in the mice with ICH, proven by worsened behavioral readouts, increased TUNEL-positive cells, and increased water content of the brain. But on day 7, the role of miR-144/451 is not obvious; this may be because miRNAs play its role when subjected to intense stimulation (13). Fu et al. proved that miR-451 mimics improved the neurological deficits induced by cerebral ischemia/reperfusion in stroke mice (19). Despite more emerging evidences showed the protective roles of miR-144/451, several other studies showed that they exaggerated brain injuries in special neurological diseases (23–26). For example, it showed that the KO of miR-451 alleviated the behavior deficits and apoptosis of hippocampus in mice with KA-induced epilepsy by upregulating GDNF level (26). This contradiction might be related with miR-144/451 targeting different genes in different pathologic conditions. Moreover, only Nissl staining is applied to detect the perihematomal edema in ICH mice in this study. T2-weighted magnetic resonance (MRI) is a better way to detect brain edema in ICH mice *in vivo*. However, the tissue loss volume would be more ambiguous compared with histology assessment (5 μm), because of the slice thickness of MRI (2.0 mm). The images from MRI cannot show clear anatomical definition of the brain area including striatum, hippocampus, and white matter structures. The future study will apply both the macroscopic timed imaging modalities and histology to measure the brain edema in different time points in ICH mice. We further detected the miR-144/451's levels in clinical trials; miR-451 decreased in sera from the patients with ICH, whereas miR-144 showed no significant change in the patients with ICH compared with the normal persons. Our preclinical and clinical data suggested that higher level of the cluster of miR-144/451 indicated a good prognosis of prediction in the patients with ICH.

Oxidative stress under ICH conditions is detrimental to neurons and deeply involved brain injuries in models or patients with ICH (1). A better understanding of the mechanisms of ICH-induced oxidative stress could offer promising strategy for treating ICH (2). As our laboratory has reported, miR-451 protects against oxidative stress in erythroid cells by targeting 14-3-3ζ which sequesters the transcription factor Foxo3 to cytoplasm and allows the transcription of two antioxidant genes, namely, cat and gpx1 (13, 27). The present observations showed that the levels of ROS and GPX produced in the mice with miR-144/451-KO ICH decreased, while MDA increased compared with that of the mice with ICH-wide type (WT), this may indicate a mechanistic interpretation for the protective effects of miR-144/451 in ICH. Furthermore, the KO of miR-144/451 in the mice with ICH reduced the antioxidant ability of neurons by activating 14-3-3ζ, thus inhibiting the transcription of cat and gpx1. The results in this study clearly show that miR-144/451 existing around the perihematomal area protects the survival neurons against oxidative stress. Consistently, Wang et al. found that the depletion of miR-144/451 promoted the ischemia/reperfusion–induced oxidative stress by activating the pathway of CUGBP2-COX-2 (28). It suggested that the cluster of miR-144/451 played a profound inhibitory effect on oxidant injuries and facilitated neuronal survive in brains of mice with ICH. However, another study demonstrated that antagomiR-451 could inhibit oxidative stress induced by OGD/R through the activation of AMPK signaling in neurons (29).

Neuroinflammation in ICH is evoked by hematoma degradation products, including the hemin, fibrin, and thrombin. The blood components promote microglia activation and the secretion of proinflammatory cytokines (1, 3). Previous studies have demonstrated that miR-451 inhibited the neuroinflammation in chronic inflammatory pain through suppressing microglia activation via targeting TLR4 (30). In our study, the KO of miR-144/451 in the mice with ICH markedly upregulated the secretions of IL-1β and TNF-α around hematomas, together with the number of GFAP and Iba1-positive neurons. These results are in line with the anti-inflammatory effects of miR-144/451 observed in the other diseases (17, 21). Chung et al. reported that the KO of miR-451 deteriorated the peribronchial inflammation in the airways of allergen-challenged mice (21). We further detected the relationship between the level of miR-451 and the levels of proinflammatory cytokines, such as IL-1β and TNF-α, and found a negative correlation between them. These results suggested that functional impairment of miR-144/451 could exaggerate human brain injuries by promoting the neuroinflammation in the patients with ICH.

FoxO3, highly expressed in brain tissue, plays crucial roles in cell proliferation, differentiation, and oxidative stress as transcription factor (30). When subjected to stimulation, FoxO3 is activated and binds to the 14-3-3 proteins in the nucleus, inhibiting the FOXO3 transcription (31). Li et al. revealed that FoxO3 has the potential to promote neuronal damage following cerebral ischemia, indicating suppression of FoxO3a may protect neurons against ischemic injury (32). Our findings illustrated that the expression of 14-3-3ζ is suppressed, whereas the level of FoxO3 increased in response to ICH injury; loss of miR-144/451 upregulated levels of both proteins. Immunostaining confirmed that ICH operation leads to FoxO3 translocating from the cytosol to nucleus; KO of miR-144/451 promoted this procession. Yu et al. reported that loss of miR-451 promoted accumulation of 14-3-3z and inhibited the activity of FoxO3 and its downstream genes including SOD and CAT (13). This different behavior of miR-144/451 might be related with the different mechanism in brain and erythrocytes.

In conclusion, we applied an easily reproduced mice model with miR-144/451 KO to show that loss of the cluster of miR-144/451 worsened the neurological function deficits and brain injuries in mice with ICH. Loss of the cluster of miR-144/451 promoted the overgeneration of ROS via activating the regulatory axis of miR-451-14-3-3ζ, which resulted in the death of neuron in the pathogenesis of ICH. Additionally, the cluster of miR-144/451 also highlighted the modulation on neuroinflammation and oxidative stress following ICH. Interestingly, we identified that miR-451 was dominant for regulating the ICH. Future studies will establish a single model with miR (-144 or−451) KO to test the exact importance of miR-144/451 in brain injuries during ICH.

## Data Availability Statement

The datasets generated for this study can be found in online repositories. The names of the repository/repositories and accession number(s) can be found in the article/supplementary material.

## Ethics Statement

The human studies were reviewed and approved by the Medical Ethics Committee of the Subei Hospital, Yangzhou, China. Written informed consent was provided by the patients for participation in the study, or where necessary, their next of kin. The animal studies were reviewed and approved by the Institutional Animal Care and Use Committee and the Animal Ethics Committee of Yangzhou University [SYXK (Su) IACUC 2017-0045].

## Author Contributions

JX, XW, and YHo conceived and designed the study. JX and DY obtained the fundings. LW and XD performed animal experiments. YHu, YS, YW, and ZD collected patients' blood samples and contributed data and analysis. CW and DY contributed generation of the manuscript. All authors contributed to the editing of the manuscript.

## Conflict of Interest

The authors declare that the research was conducted in the absence of any commercial or financial relationships that could be construed as a potential conflict of interest.
